# Social Economic Costs, Health-Related Quality of Life and Disability in Patients with Cri Du Chat Syndrome

**DOI:** 10.3390/ijerph17165951

**Published:** 2020-08-17

**Authors:** Yllka Kodra, Marianna Cavazza, Marta de Santis, Andrea Guala, Maria-Elena Liverani, Patrizio Armeni, Maura Masini, Domenica Taruscio

**Affiliations:** 1 National Centre for Rare Diseases, Istituto Superiore di Sanità, 00161 Rome, Italy; marta.desantis@iss.it (M.d.S.); domenica.taruscio@iss.it (D.T.); 2 Centre for Research on Health and Social Care Management (Cergas), SDA Bocconi School of Management, 20136 Milan, Italy; marianna.cavazza@unibocconi.it; 3 Paediatric Unit, Castelli Hospital, 28922 Verbania, Italy; andrea.guala@aslvco.it; 4 Pedriatic Unit, Sant’Andrea Hospital, 00189 Rome, Italy; mliverani@ospedalesantandrea.it; 5 Center for Research in Health and Social Care Management, Bocconi University, 20136 Milan, Italy; patrizio.armeni@unibocconi.it; 6 ABC, Cri du Chat patients Association, 50026 Florence, Italy; restauro@mauramasini.it

**Keywords:** Cri du Chat syndrome, quality of life, disability, cost of illness, rare diseases

## Abstract

*Background*: Cri du Chat syndrome (CdC) is a rare disease caused by the deletion on the short arm of the chromosome 5, with an incidence of 1:15,000 to 1:50,000 live-born infants. No study at international level has assessed the costs, Quality of Life (QoL) and Disability through standardized quantitative tools. The aim is to estimate economic costs related to CdC from a societal perspective, to assess the QoL and Disability in patients with CdC along with their caregivers in Italy. *Methods*: A cross-sectional study of patients with Cri du Chat in Italy was carried out. A cost of illness approach from a societal perspective was used to estimate cost, and a micro-costing method was adopted. The QoL was measured with EuroQol 5-domain (EQ-5D) questionnaire and Disability by using World Health Organization Disability Assessment Schedule 36 item (WHODAS 2.0). *Results*: A total of 76 questionnaires were collected from caregivers taking care of 40 adult patients and 36 minor patients. All patients need a carer and the principal caregiver is commonly informal carer or a family member (93%). The EQ-5D VAS score for patients is 65.5 (SD = 22.4) out of 100; while the most important compromised areas of QoL are usual activities and self-care. The overall WHODAS 2.0 score is 65% (0 = no disability; 100 = full disability). The average annual cost of a patient with Cri du Chat in our population is €87,856.24; the main cost item of patients with Cri du Chat syndrome is informal care (i.e., €76,981.69 yearly) since it constitutes the 87% of total costs. Results highlight the burden of CdC in terms of its impact on QoL and Disability for patients and caregivers in Italy, with a score much lower than that of general population. The disease is associated with considerable costs of informal care. *Conclusions*: Cri du Chat syndrome was found to be linked with a significant socioeconomic impact which is dominated by direct non-healthcare informal costs.

## 1. Introduction

Cri du Chat syndrome (OMIM 123450, ORPHA281) is a rare disease caused by total or partial deletion on the short arm of the chromosome 5 (5p-). The clinical picture, severity, and progression of the disease varies depending on the region of the chromosome that is deleted and whether it is terminal or interstitial as well as its size (88% to 90% of cases result from terminal deletions of chromosome 5 while 3% to 5% are due to an interstitial deletion) [1,2]. In other words, differences in phenotype can be attributed to the differences in genotype, associated with an extensive clinical phenotype heterogeneity and with neurodevelopmental abnormalities.

The incidence ranges from 1:15,000 to 1:50,000 live-born infants [3]. Main clinical features include high-pitched cry, microcephaly, broad nasal bridge, epicanthal folds, micrognathia, and severe psychomotor retardation. Cardiac and renal malformations may be also found [4]. There is no specific treatment for CdC nevertheless, patients benefit from rehabilitative programs, which should be started as soon as possible and involve close collaboration with families, who must be supported psychologically [5]. Moreover, it is required that clinical evaluations take place regularly over the course of a person’s life, more specifically from the neonatal to adulthood period [6]. At our knowledge, no study at Italian, nor at the international level has assessed and estimated the costs, the impact of Cri du Chat syndrome on Quality of Life (QoL) and disability through standardized quantitative tools. The aim of the study is to estimate social and economic costs and to assess the QoL and disability in patients with Cri du Chat and their caregivers in Italy.

## 2. Materials and Methods

This was a cross-sectional study of people diagnosed with Cri du Chat recruited from the Italian Cri du Chat Association (ABC Associazione Bambini Cri du Chat Onlus, Florence, Italy: www.criduchat.it). The study was carried out between May and September 2017. The study was approved by the Scientific Committee and the Review Board of the Cri du Chat Association and each subject gave informed consent.

The survey was completely anonymous, as the patients were contacted by their patient organization and their responses without any identification data were sent directly to the researchers. As the degree of mental retardation is significant, for all patients (adult ≥ 18 years and minor < 18 years), the questionnaires were completed by a parent or other caregiver.

Information were collected by questionnaires administered by e-mail through patient association. The response rate of the survey, after one follow-up mailing, was 64% (emails sent to a total of 118 caregivers; number of respondents was 76).

The questionnaires included questions regarding patients’ socio-demographics characteristics (age, gender, education level, marital status, employment, etc.), use of healthcare services and goods covering the 6-month period prior to the study (12 months for hospital admissions and specialist medical visits; 1 months for drugs), impact of the diseases on working hours and conditions loss and social burden (temporary and permanent sick leave or early retirement) and presence of any formal or informal care (Supplementary File 1).

Caregivers were also asked to complete the WHODAS II, for disability assessment (Supplementary File 2). They also completed the EuroQol 5-domain (EQ-5D) to rate their Quality of Life (Supplementary File 3) and also their subjective perception of QoL of their children with Cri du Chat (Supplementary File 4). Moreover, the patient’s principal caregivers were asked to answer a separate questionnaire detailing their characteristics and time spent caring for the person with Cri du Chat (Supplementary File 5).

A pilot testing of the questionnaire to a focus group of 10 caregivers was performed before data collection began. The objective was to ensure that the questions being asked accurately reflect the information the researcher desired and that the respondent could and wanted to answer the questions.

### 2.1. Patient and Caregiver Outcomes

Patient and caregiver outcomes, in terms of health-related quality of life (HRQoL) and Disability were obtained respectively by means of self-administered EQ-5D questionnaire and WHO Disability Assessment Schedule (WHODAS II).

The EQ-5D questionnaire is a simple generic instrument developed by a multidisciplinary group of researchers [7]. This questionnaire has been validated in many countries in Europe, and is commonly used in economic evaluation and technology assessment. There are five dimensions in the EQ-5D which cover the areas of mobility, self-care, everyday activities, pain/discomfort and anxiety/depression.

Each of the five dimensions comprising the EQ-5D descriptive system is divided into 5 levels of perceived problems: Level 1: indicating no problem; Level 2: indicating slight problems; Level 3: indicating moderate problems; Level 4: indicating severe problems; Level 5: indicating extreme problems. Evaluation of HRQoL has been reported for patients and caregivers.

The EQ-5D-5L Proxy version was used in the study, as Cri du Chat patients are mentally or physically not capable reporting on their health-related quality of life, for instance because of severe intellectual disability or mental health problems. The caregiver (the proxy) is asked to rate how he/she thinks the patient would rate his/her own health-related quality of life.

The WHO Disability Assessment Schedule (WHODAS II), 36-Item Interviewer Administered translated Italian version, was used for the assessment of disability [8]. It is a generic measure, it can be used within and across disorders to determine the relative impact of the disorder.

The scale contains 36 items on functioning and disability, covering six domains of functioning during the last 30 days:

Cognition—understanding & communicating
Mobility—moving & getting around
Self-care—hygiene, dressing, eating & staying alone
Getting along—interacting with other people
Life activities—domestic responsibilities, leisure, work & school
Participation—joining in community activities.

Cognition Domain I (Understanding and Communicating) consisted of six questions about cognitive abilities in communication with others. Domain II (Mobility) contained five questions about the difficulties faced during moving and getting around. Domain III (Self-care) incorporated four questions about troubles faced during eating, washing and dressing. Domain IV (Getting along with people) comprised five questions about how much difficulty the visually impaired faced in dealing with people and keeping friendships. Domain V (Life activities) included eight questions about the difficulties experienced in performing their household responsibilities and studying. The last domain (Participation in society), included eight questions asking about the participation in society and the impact of the disability on themselves and the family.

All the questions in the six domains were measured on a 5-point Likert scale ranging from: “none difficulties” (1), “mild difficulties” (2), “moderate difficulties (3), “severe difficulties” (4), and “extreme difficulties” (5). Raw scores are transformed into standardized scores, with 0 indicating the highest level of functioning and 100 indicating the lowest level of functioning. Scores were calculated for the separate domains and all domains combined. Subsequently, the scores were converted into a metric ranging from 0 to 100 where higher scores reflect greater disability.

If participants did not work, only 32 items were administered and the life activities score is based only on participation in home-related activities. Because most of the study participants were retired, the 32-item version was used in analyses. An average domain score and a general disability score were produced.

### 2.2. Costing Methodology

**Figure 1:**
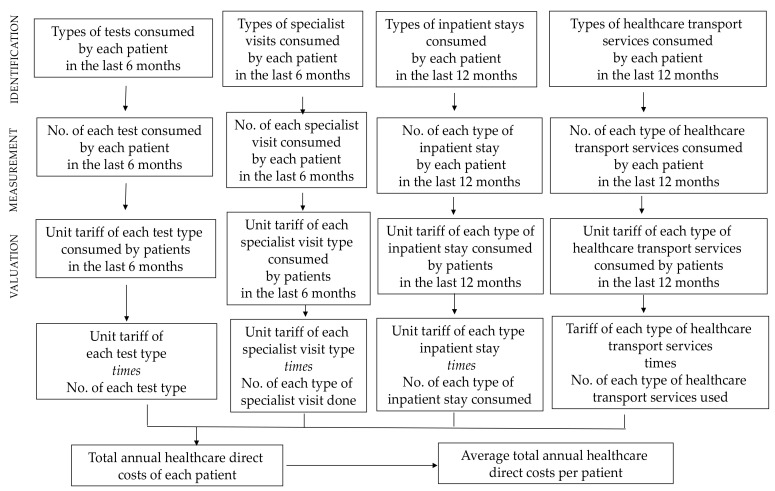
Description of the procedure implemented to estimate the average direct healthcare cost per patient according to micro-costing method.

We measured the economic burden of CdC syndrome in our population, referring to the scientific literature about rare diseases’ costs, adopting a cost-of-illness approach from a societal perspective [9]. Two key instances are the CHESS (Cost of Haemophilia in Europe: A Socioeconomic Survey) [10] and BURQoL-RD (Social economic BURden and health-related Quality of Life in patients with Rare Diseases in Europe) projects [11]. Specifically, in this second case, the project’s goal was to develop a disease-based model quantifying the socio-economic burden and HRQOL of patients suffering from ten rare diseases and their caregivers in nine European countries. The common method used in these studies [12,13,14] to estimate costs was micro-costing. Micro-costing implies the ‘direct enumeration and costing out of every input consumed in the treatment of a particular patient’ [15]. Actually, micro-costing method allows estimating effectively the true costs borne by both the healthcare system and society [16,17]. The three phases of micro-costing are: identification, measurement and valuation. The first phase requires the identification of all the relevant resources mobilized by patients (cost items) and their classification into direct healthcare costs, direct non-healthcare costs and indirect costs. Consistently with the societal perspective, we included not only healthcare services and drugs, but also use of formal and informal care and productivity loss by caregivers along with non-healthcare resource consumption (e.g., transportation, etc.). The measurement phase requires the quantification of the amount of each cost item mobilized for each patient over the observation period. Finally, the valuation phase requires the identification of unit costs associated to each cost item to be multiplied by the mobilized quantity. At the end of these three phases, we obtained per-patient costs for each cost item (Figure 1) and we were able to estimate the average cost per patient in one year with 2017 as the reference year.

For the valuation phase, we have firstly considered direct health care costs including drugs, medical tests, medical visits, hospitalizations, medical devices and health care transport. In this case, we were required to use tariffs instead of costs because a national healthcare services’ costs data set is not available in Italy. Moreover, at the moment, outpatient services’ national tariffs have not been updated in the last twenty-five years and regional tariffs are the main actual reference in Italian NHS. Hence, we have applied those set by Region Lombardy which update nearly regularly its tariffs according to costs surveys and they represent a reference point at national level. Concerning hospital inpatient stays, we could apply national tariffs recently updated. Moreover, we estimated the healthcare services fees out of Italian National Health Service, paid out of pocket by patients: we selected a small-middle and a big town, adopting a 250,000 inhabitants cut-off provided by the Italian association of city-councils [18,19] in Northern, Centre and Southern Italy and then we ran a pricing market analysis involving several providers. Last, we did not value drugs because patients did not consume any medicine related to CdC possible symptoms (i.e., cardiac or renal diseases) and there is not any specific pharmaceutical treatment for this disease.

Direct non-healthcare costs include formal and informal care. Specifically, direct non-healthcare formal costs comprise formal care provided by professionals and social healthcare and social services managed at city-council level. This type of service is characterized by high fragmentation in their provision, which led us to implement again the above described strategy to collect information on these cost items without national or regional standards. Direct non-healthcare informal costs cover informal care provided by relatives and friends rather than by contracted professionals. In this case, we have considered if the informal caregiver had not provided these services, he/she would need to be replaced by another person who could provide the necessary care, applying the average hour wage of contracted professionals [20,21,22]. The administered questionnaire surveyed also the total amount of hours of provided informal care; however, as a conservative criterion, and preventing joint production, we have censored the time of care to a maximum of 16 h per day (112 h per week) when the time of care reported exceeded this figure.

Last, indirect costs refer to the output lost by society because of cessation or reduction of productivity (i.e., productivity loss) resulting from morbidity, or disability brought on by the disease. Collected information about patients and caregivers through the questionnaire were (i) the working days and the hours per day lost because of disease and its care, and (ii) the age of their possible early retirement. In this way, we have estimated the productivity loss of both patients and caregivers, considering both the sick leaves of both patient and caregiver as well as the economic impact of an early retirement. Last, we have applied the human capital approach [23,24], valuing the amounts of days and hours of sick leaves by the per hour Italian average wage, according to 2017 Eurostat estimation.

## 3. Results

During 2017, a total of 76 questionnaires were collected from caregivers taking care of 40 adult patients (≥18 year) and 36 children (<18 year) with Cri du Chat syndrome. The mean age of all patients was 19,6 years (mean = 28 years for adults and mean = 10 years for children and the majority was male (68%); all patients needed a caregiver to assist their daily activities. The principal caregiver was a family member (93%).

### 3.1. Patients’ Health Related Quality of Life

HRQoL data was available for 60 patients, 33 adults (82,5% of the adult population) and 27 children (75%).

In Table 1, the percentage of reported problems (the sum of the proportion of reported levels 2 to 5 for each of the five EQ-5D-5L) for each dimension, divided by adult and children, are presented. For each dimension, non-statistical differences were found between adults and children.

The most important compromised areas of QoL are (1) usual activities (100% reported problems in performing usual activities such as work, study, housework, family or leisure activities), (2) self-care (98% reported problem in washing and dressing) and (3) mobility (92% reported problem in walking). Slight and moderate problem is stated by caregivers for patient anxiety (38%) and pain (55%) dimensions.

### 3.2. Caregivers’ Health Related Quality of Life

A total of 56 principal caregivers (74%) answered to the questionnaire concerning quality of life. The characteristics of the study caregivers are reported in Table 2.

**Figure 2:**
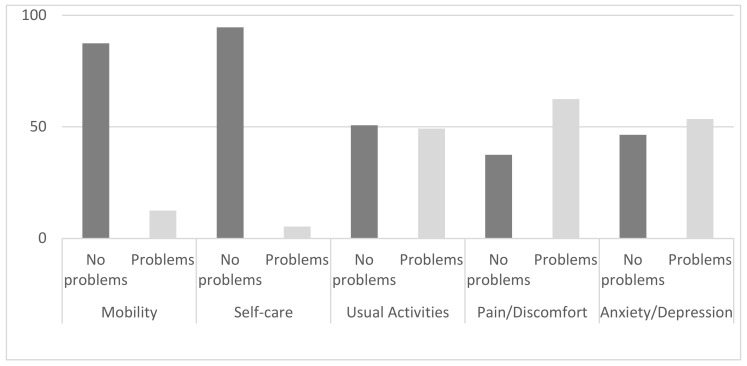
The percentage of reported problems for each dimensions of caregivers QoL.

The main principal caregiver of patients is a family member (93.4%); only 4 persons were professionals. In Figure 2, the percentage of reported problems for each dimension of caregivers QoL are presented.

With regards to EQ-5D VAS the patients’ score was statistically lower (*p* < 0.001) than caregivers (respectively 65.5 (ES = 2.9) out of 100 for patients versus 74.6% (ES = 2.2) for caregivers) (Table 3).

### 3.3. Patient’s Disability Assessment

The questionnaire of disability was available for 33 adult patients. The overall WHODAS II score was 65% (where 0 = no disability; 100 = full disability), with higher score of disability for Life activities (87%), Understanding and communicating (73%) and Getting along (62%). Table 4 shows the baseline scores (mean and SD) from the six domains’ outcome measures.

### 3.4. Patient’s Costs

The average annual cost per patient with Cri du Chat is €87,856.24 (Table 5). Actually, the population differentiation between children and adults shows a substantial difference in terms of costs: this value increases to €140,637.30 considering only children population and it decreases to €40,353.25 in the case of adults.

**Figure 3:**
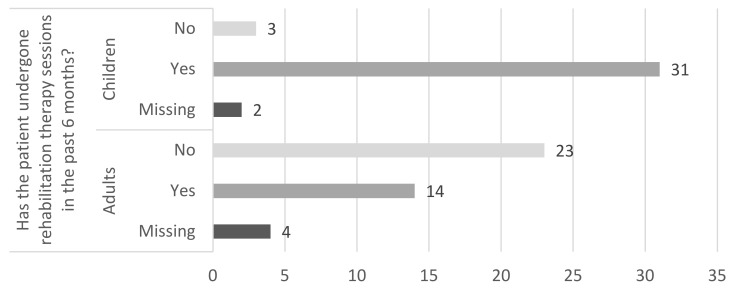
Distribution of patients undergoing rehabilitation in the six months before the survey.

While considering direct healthcare cost items, we did not include drugs as caregivers reported products prescribed for health problems not related to Cri du Chat syndrome. This result is due to the absence of any specific pharmaceutical treatment addressing this disease or its related symptoms. Therefore, the most substantial cost items concerning healthcare are hospitalizations, casualties, as well as nurse care, and rehabilitation. This latest intervention constitutes the main treatment in addressing symptoms of patients with Cri du Chat syndrome and it includes physical, cognitive and logopedic rehabilitation. Even if this item’s contribution to total costs is proportionally the same in both two subpopulations, the rate of adult patients undergoing rehabilitation is inherently different from the one with exclusively children (Figure 3).

**Figure 4:**
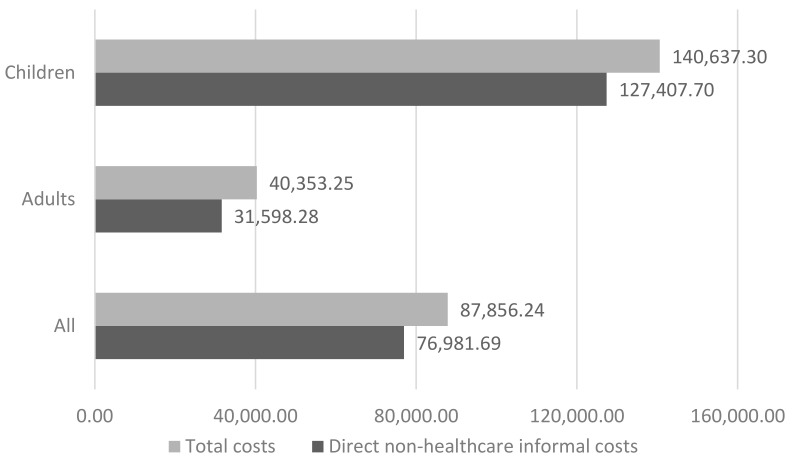
Direct non-healthcare costs related to informal care and total costs in children, adults and all patients (2017, €).

Concerning direct non-healthcare costs, data show that adults consume social care services (e.g., day-care centres or occupational centres) while this type of consumption is nearly absent in children population. Formal care provided by professionals is a very rare type of intervention as only 4 patients out of 76 reported it; however, responders did not provide details about the hours amount preventing this item inclusion in costs’ analysis. Last, the main cost item of patients with Cri du Chat syndrome is informal care (i.e., €76,981.69 yearly) since it constitutes the 87% of total costs for all patients (Figure 4).

Ultimately, indirect costs amount to a yearly average of €5083.58 and they include two main items: productivity loss and early retirement. Productivity loss is related to the working time lost by caregivers because of their activity of informal care and it concerns only adults’ caregivers. The second item, the most substantial one, is named early retirement and it makes reference to both early retired caregivers and adult patients.

As previous results show, there is a relevant difference between adults’ costs and those of children: therefore, we have tested whether these differences are statically significant or not. Results highlight that total costs of children are significantly different from those of adults and most substantial cost items, related to the key activities of care (i.e., rehabilitation and informal care), are those characterized by a statistically significant difference (Table 6).

Last, we tested also a possible correlation between costs’ level and quality of life rate, but we did not get any significant result.

## 4. Discussion

Cri du Chat syndrome is considered a complex rare disorder with a broad range of clinical behavioural/psychological characteristics associated with severe/profound intellectual disabilities and cognitive impairments and stereotyped behaviour problems [25]. The severity of the symptoms is linked to the size of deleted chromosomal region. These patterns change over time across the entire life course [26].

We used a cross-sectional survey to estimate related costs from a societal perspective using the cost of illness approach. Previous research projects like BURQOL-RD helped to critically assess the state-of-the-art issues around rare diseases and elaborate strategic tool based on the same methodology used in this study [11].

This is the first study to estimate the burden of Cri du Chat syndrome. To our knowledge, there is currently no study on the costs of Cri du Chat syndrome; QoL and disability of Cri du Chat syndrome patients has never been measured with standardized instruments.

By analysing healthcare (4.3% of the total cost of illness), non-healthcare (93.2% of the total cost of illness) and loss of labour productivity (2.5% of the total cost of illness) costs separately, we found that non healthcare costs were the largest expenditure. Direct healthcare costs confirm how Cri du Chat patients require two main types of healthcare service particularly hospitalization and rehabilitation. Moreover, our data suggest that rehabilitation programmes are provided to a great extent to children, but once adults a relevant part of them do not continue this type of treatment. Results mirror the key aspects of Cri du Chat syndrome: a delayed development, and intellectual disability requiring rehabilitation, hospital controls and a continuous necessity of care [27].

The present study quantifies the activity of informal caregivers (family members) and highlights the importance of informal care. The results confirm that that the main cost driver in Italy is informal care (paid professional), usually provided by relatives, along with their early retirements and disability support allowances supplied to adult patients. The high costs of informal care underpin the social economic burden of Cri du Chat syndrome, accounting for the largest part of total costs (87%). Hence, our costs’ analysis suggests to possible policy-makers the relevance of the adopted perspective and the necessity to support families in taking care of their relatives with Cri du Chat syndrome.

Decisively, it is worth stressing that in the case of Cri du Chat syndrome, as most part of rare diseases, only the community perspective allows to capture the real economic impact of these patients’ care. Actually, if we had adopted the NHS perspective, we would have missed the main cost item of these patients’ care.

QOL is another source of information that helps to define the impact on society of a specific health problem. Knowledge of QOL is also needed to measure the effectiveness of health interventions on disease management. Therefore, QOL can be a useful indicator together with other epidemiological indicators such as incidence, prevalence, mortality and costs to set priorities and allocate healthcare and social resources. The QOL of patients and their caregivers was also studied using standard questionnaire.

In terms of patients’ disability, all areas of evaluation were affected. In particular, 87% of patients with Cri du Chat syndrome was unable to perform life activities such as domestic responsibilities, leisure, work & school, 73% reported disability in Cognition aspect (understanding and communicating). Despite the lack of curative treatment for people affected by Cri du Chat syndrome, supportive treatments including rehabilitative can be effective for the autonomy and QoL. Also, in terms of QoL, 100% reported problems in performing usual activities such as work, study, housework, family or leisure activities; 98% reported problems in self-care such as washing and dressing and 92% reported problem in walking. In Italy, mean VAS score for Cri du Chat patients was lower than that measured in a sample representative of the Italian general population [28].

These results indicated that a great proportion of patients need help from others to perform every day activities. The principal caregivers were all family members (informal care) of the patients.

Therefore, the informal care is a major challenge. This task is even more complex if pediatric patients are involved as it is difficult to differentiate between age-related and disease-related needs.

Informal care form is often seen as a cost-effective way of preventing institutionalisation and enabling patients to remain at home. Informal care is likely to become even more important in the field of rare diseases. In Italy, the family had traditionally a strong role, probably due to largely underdeveloped formal care systems at national level. On the other hand, it is widely recognized that family involvement in educational and rehabilitation programs allows to achieve the best psychomotor, linguistic and relational development of the child. The findings suggest that formalising informal care through cash payments, legal rights, social security, and training opportunities can have important beneficial effects on informal carers and the patients that they care for [29]. The lack of correlation between economic and social burden of families and patients’ quality of life, tested in other diseases [30], may be due to a missing resolutive treatment for patients with CdC syndrome. Hence, it seems there is not a proportionality between families’ investment in terms of resources and care and the related outcome.

Research focused on families of patients affected by CdC is very limited [31]. The complexity of the clinical manifestations and its requirement for supportive treatment interventions as well as the continuous monitoring and care explain the significant impact in terms of cost as well the considerable effect on quality of life. The presence of behaviour problems related to the disorders [32] are predictor and determinant of familial stress for parents of a child with CdC [33].

Some limitations of the study have to be mentioned. The recruitment of patients was carried out by patient organizations and patients with no contact with patient organizations could therefore not be invited to participate in the study. Another limitation of the study was that no data on disease severity, CdC genotype and/or phenotypic classification was collected by the questionnaire. These measures could be assessed by clinicians which were not involved in our study. As CdC is a rare disease with a wide range of clinical and severity variability due to the region of the chromosome that is deleted, it is important to consider these clinical measures in the future research study in order to permit their effects on HRQOL and cost calculation. The design of the study was cross-sectional and the information was retrospectively collected leading to recall bias.

As children or adult affected by CdC syndrome are mentally or physically incapable for filling the different study questionnaires, because of severe intellectual disability or mental health problems, caregivers were asked to rate the patient’s Qol and disability in their (the proxy’s) opinion. It is known that children and parents are likely to have different perspectives of the children’s quality of life: parents tend to rate their child’s quality of life lower than the child’s own ratings, especially in domains that are more subjective than objective. Nevertheless, there is a growing tendency to include parent’s perspectives to assess outcomes of their disabled children.

## 5. Conclusions

Cri du Chat syndrome was found to be linked with a significant socioeconomic impact which is dominated by non-healthcare costs related to informal care: hence, the management of this disease is an expensive and demanding undertaking for patients’ families. It is imperative that these problems are effectively addressed and managed by regional as well as national authorities through specific public health plans and strategies, and investing more, resources in long term care services. Socio-economic, QoL and disability data can be essential indicators in order to characterize the burden of rare diseases, to set priorities and to assess the outcomes of health policies. Additionally, they are also impactful in new interventions for both clinicians and health policy decision-makers at the local, regional, national or international level. In conclusion more research is required to progress in this field as shown by our study on patients with Cri du Chat syndrome.

## Figures and Tables

**Table 1:** The percentage of reported problems for each QoL dimensions divided by adult and children.

		Adult	Children	Total
Mobility	No problems	12.1%	3.7%	8.3%
	Problems	87.9%	96.3%	91.7%
Self-care	No problems	3.0%	0.0%	1.7%
	Problems	97.0%	100.0%	98.3%
Usual activity	No problems	0.0%	0.0%	0.0%
	Problems	100.0%	100.0%	100.0%
Pain/Discomfort	No problems	36.4%	44.4%	40.0%
	Problems	63.6%	55.6%	60.0%
Anxiety/depression	No problems	51.5%	70.4%	60.0%
	Problems	48.5%	29.6%	40.0%

**Table 2:** Characteristics of the study caregivers.

No. of Responses	56
Mean age, years (SD)	50.0 (13.3)
Female (%)	85.7
Married (%)	75
**The principal caregivers**	
Informal (family member) %	93.4
Formal (paid professional)	5.1
Mean number of members of a patient’s family (min-max)	2.9 (1–6)
**Relationship to patient**	
mother/father (%)	92
sister/brother (%)	6
**Employment situation**	
housewife (%)	33.9
workers	37.5
retired (%)	14.3
withdrawn from professional activities	12.5
other	1.8

**Table 3:** EQ 5D VAS value for patients and caregivers.

EQ VAS	Patients	Caregivers
Mean	65.5	74.6
ES	2.9	2.2
25th	50	64
75th	85	85

**Table 4:** WHODAS II value for adult patients with Cri du Chat syndrome.

	Mean (SE)	Median (25th–75th)
Cognition (understanding & communicating)	73.6 (4.2)	83.3 (62.5–91.7)
Mobility (moving & getting around)	37.9 (6.4)	25.0 (0.0–70.0)
Self-care (hygiene, dressing, eating & staying alone)	48.8 (4.2)	52.0 (40.0–60.0)
Getting along (interacting with other people)	61.5 (4.6)	60.0 (45.0–82.0)
Life activities (domestic responsibilities, leisure, work & school)	87.1 (3.3)	100.0 (79.7–100.0)
Participation (joining in community activities)	58.9 (3.9)	59.3 (40.6–75.0)

**Table 5:** Average annual costs per patient with Cri du Chat syndrome—All, adults and children (2017, €).

	ALL		ADULT		CHILDREN	
	Mean	SD	Mean	SD	Mean	SD
**Direct costs (Direct Healthcare Costs & Direct Non-Healthcare costs)**	**€82,782.60**	**€105,359.30**	**€36,282.45**	**€67,134.65**	**€134,449.40**	**€116,395.50**
***Direct health care costs***	***€4271.06***	***€6845.92***	***€2259.59***	***€3615.74***	***€6506.03***	***€8723.47***
Medical tests	€58.47	€257.97	€30.91	€128.92	€89.09	€349.64
Medical visits	€481.26	€928.49	€346.03	€680.17	€631.50	€1134.78
Hospitalizations, casualty, nurse care	€1405.73	€5375.17	€827.89	€2206.76	€2047.78	€7461.93
Rehabilitation	€1996.03	€3920.79	€746.33	€1765.31	€3384.58	€5069.59
Health material	€293.86	€660.76	€251.04	€553.68	€341.43	€767.83
Healthcare transport	€39.93	€112.06	€65.40	€141.40	€11.64	€55.38
***Direct non-healthcare formal costs***	***€1529.85***	***€7258.12***	***€2424.58***	***€9935.55***	***€535.70***	***€978.66***
Non-Healthcare transport	€246.82	€362.95	€201.33	€266.51	€297.37	€445.14
Social care	€1283.03	€7247.90	€2223.25	€9930.75	€238.33	€738.33
***Direct non-healthcare informal costs***	***€76,981.69***	***€103,146.50***	***€31,598.28***	***€65,184.63***	***€127,407.70***	***€114,430.50***
Main informal care	€48,889.33	€63,619.13	€21,686.81	€47,039.45	€79,114.35	€66,498.53
Other informal care	€28,092.36	€48,314.80	€9911.48	€24,958.70	€48,293.35	€59,249.30
***Indirect costs***	***€5083.58***	***€1354.56***	***€4092.15***	***€1183.33***	***€6185.16***	***€-***
Productivity loss	€7.30	€63.66	€13.88	€87.75	€-	€-
Early retirement	€5076.28	€1356.71	€4078.28	€1176.07	€6185.16	€-
**TOTAL COSTS**	**€87,856.24**	**€105,916.30**	**€40,353.25**	**€67,301.99**	**€140,637.30**	**€116,394.10**

**Table 6:** Two-tailed t test with equal variances testing whether the cost differences between adults and children are statistically significant.

Cost Items	Null Hypothesis: Difference Between Adults and Children Costs = 0
**Direct costs**	Pr(|T| > |t|) = 0.0000 ***
***Direct healthcare cost***	Pr(|T| > |t|) = 0.0061 ***
Medical tests	Pr(|T| > |t|) = 0.3295
Medical visits	Pr(|T| > |t|) = 0.1826
Hospitalizations, casualty, nurse care	Pr(|T| > |t|) = 0.3265
Rehabilitation	Pr(|T| > |t|) = 0.0028 ***
Healthcare materials	Pr(|T| > |t|) = 0.5551
Healthcare transport	Pr(|T| > |t|) = 0.0359 *
***Direct non healthcare formal costs***	Pr(|T| > |t|) = 0.2600
No healthcare transport	Pr(|T| > |t|) = 0.2521
Social care	Pr(|T| > |t|) = 0.2357
***Direct non healthcare informal costs***	Pr(|T| > |t|) = 0.0000 ***
Main informal care	Pr(|T| > |t|) = 0.0000 ***
Other informal care	Pr(|T| > |t|) = 0.0004 ***
***Indirect costs***	Pr(|T| > |t|) = 0.0000 ***
Productivity loss	Pr(|T| > |t|) = 0.3462
Early retirement	Pr(|T| > |t|) = 0.0000 ***
TOTAL COSTS	Pr(|T| > |t|) = 0.0000 ***

*** difference is highly significant; * difference is slightly significant.

## Supplementary Materials

The following are available online at https://www.mdpi.com/1660-4601/17/16/5951/s1, Supplementary File 1: Patient questionnaire; Supplementary File 2: WHODAS II questionnaire; Supplementary File 3: Caregiver EQ-5D questionnaire; Supplementary File 4: Children EQ-5D questionnaire; Supplementary File 5: Caregiver questionnaire.
