# Supplementary material for: Social Economic Costs, Health-Related Quality of Life and Disability in Patients with Cri Du Chat Syndrome
Source: Int J Environ Res Public Health. 2020 Aug 17;17(16):5951. doi: 10.3390/ijerph17165951 (PMC7459640; doi:10.3390/ijerph17165951)
Supplement: Supplementary file 1 [file ijerph-17-05951-s001.zip › S1.pdf]

# Questionario - Pazienti da 18 o più anni

***Il questionario deve essere compilato dalla persona responsabile della cura del paziente.***

**1. Quale tipo di relazione ha con il paziente? Lei è:**

o Madre / Padre

o Zio / Zia

o Fratello / Sorella

o Tutore legale

o Persona che assiste il paziente su base professionale

o Altro (per favore, specificare) _________

**2. Età del paziente** _____

**3. Sesso del paziente**

o Femmina　　　o Maschio

**4. Provincia di residenza del paziente** _______________

**5. Stato civile del paziente**

o Celibe/Nubile

o Coniugato/a o convivente

o Divorziato/a

o Separato/a

o Vedovo/a

**6. Titolo di studio del paziente**

o Diploma di scuola media inferiore

o Attestato di frequenza di scuola media superiore

o Diploma di scuola media superiore

• o Laurea universitaria

• o Nessuno

**7. Numero di membri del nucleo familiare in cui il paziente vive:**

N° ___ di persone escluso il paziente

**8. A quale età è stata diagnosticata al paziente la malattia?** ___ anni

**9. Il paziente ha bisogno di una persona per assisterlo nelle attività quotidiane?**
*(ad es. igiene personale, aiuto nel muoversi, somministrazione farmaci, svolgimento dei trattamenti terapeutici, ecc. ecc.)*

o Sì　　　o No ⇒ *si passi alla domanda 12*

**10. Se sì, chi è la persona principale che assiste il paziente?**

o Un membro della famiglia ⇒ si passi alla domanda 12

o Una persona non su base contrattuale (ad es. amico o volontario) ⇒ si passi alla domanda 12

o Una persona su base professionale e contrattuale (assunto direttamente con contratto o fornito da un ente di assistenza)

**11. Se sono utilizzati i servizi di assistenza forniti da una persona su base contrattuale**

a. Per quante ore alla settimana? ______ ore alla settimana

b. Chi paga il servizio e quanto?

o La persona responsabile della cura del paziente paga l'intero costo di ______ € per ora

o Il costo è coperto totalmente da enti pubblici

o Il costo è parzialmente coperto da enti pubblici, per cui la persona responsabile della cura del paziente paga ____ € per ora

**12.Qual è la sua situazione lavorativa del paziente?**

o Occupato

o Studente ⇒ *Si passi alla domanda 19*

o Disoccupato ⇒ *Si passi alla domanda 15*

o Congedo temporaneo ⇒ *Si passi alla domanda 15*

o Stato di invalido permanente ⇒ *Si passi alla domanda 17*

o Pensionato ⇒ *Si passi alla domanda 17*

o Casalinga ⇒ *Si passi alla domanda 19*

o Collocamento mirato

**13. Se il paziente è occupato, specificare il tipo di contratto**

o Tempo determinato

o Tempo indeterminato

o CoCoPro

o Vaucher

o Altro (specificare)_____________________________________________

**14.Se il paziente è occupato, specificare la retribuzione mensile (importo al netto)** __________ €

**15.La malattia ha comportato al paziente problemi nello svolgimento della sua attività lavorativa in questi ultimi 6 mesi?**

*(Rispondere solo se il paziente è occupato, disoccupato o è in congedo temporaneo)*

o Sì o No ⇒ *Si passi alla domanda 19*

**16. Se sì, per favore specifichi quali:**

o Il paziente è stato assente dal lavoro per ___ giorni

o Il paziente ha lavorato ___ ore in meno al giorno per ___ giorni

o Il paziente sta lavorando ___ ore in meno al giorno

o Il paziente non lavora meno ore al giorno, ma ha problemi nello svolgimento del suo lavoro

o Altri problemi (per favore specificare): ____________________________________

**17.Il paziente ha deciso di lasciare il lavoro o di andare in pensione anticipata a causa della sua malattia?**
*(Rispondere solo se il paziente è invalido permanente o pensionato)*

o Sì

o No ⇒ *Passi alla domanda 19*

o Non è stato più in grado di lavorare in seguito alla sua malattia ⇒ *Passi alla domanda 19*

**18. Se sì, per favore specifichi con quali modalità**

o Il paziente ha dovuto lasciare il lavoro all'età di ___ anni

o Il paziente ha dovuto andare in pensione anticipata all'età di ___ anni

**19.Il paziente ha un certificato di invalidità con la relativa percentuale?**

o Sì, ce l'ha - Per favore, indichi la percentuale riconosciuta: ____ %

o No

**20. Il paziente ha un assegno di invalidità?**

o Sì - Per favore, specifichi da quanti anni ____

o No

**21. Il paziente ha assunto dei farmaci nell'ultimo mese?**

o Sì

o No ⇒ *Passi alla domanda 23*

**22.Quali farmaci il paziente ha assunto nell'ultimo mese sia a domicilio sia in ospedale (specificatamente a causa della sua malattia)?**

Per favore, segni i farmaci assunti e specifichi la forma di pagamento

| | Nome commerciale del farmaco (o principio attivo) | Costo coperto dal Servizio Sanitario Nazionale (ASL) | | |
|---|---|---|---|---|
| | | Sì | No | Parziale |
| o | | o | o | o |
| o | | o | o | o |
| o | | o | o | o |
| o | | o | o | o |
| o | | o | o | o |
| o | | o | o | o |
| o | | o | o | o |
| o | | o | o | o |
| o | | o | o | o |

**23. Negli ultimi 6 mesi, il paziente ha fatto test o esami medici, prescritti da un medico (specificatamente a causa della sua malattia)?**

o Sì

o No ⇒ *Passi alla domanda 25*

**24.Quali test o esami medici, prescritti da un medico, il paziente ha fatto negli ultimi <u>6 mesi</u> (specificatamente a causa della malattia)?**

*Se uno dei test non è elencato, per favore lo specifichi.*

| | Test ed esami medici | N° di volte in 6 mesi | Costo coperto dal Servizio Sanitario Nazionale (ASL) | | |
|---|---|---|---|---|---|
| | | | Sì | No | Parziale |
| o | Esami del sangue | | o | o | o |
| o | Esame delle urine | | o | o | o |
| o | Radiografia | | o | o | o |
| o | Esame dell'udito (Audiometria) | | o | o | o |
| o | Elettrocardiogramma | | o | o | o |
| o | Ecocardiogramma | | o | o | o |
| o | Risonanza magnetica | | o | o | o |
| o | TAC | | o | o | o |
| o | | | o | o | o |
| o | | | o | o | o |
| o | | | o | o | o |
| o | | | o | o | o |
| o | | | o | o | o |
| o | | | o | o | o |
| o | | | o | o | o |
| o | | | o | o | o |
| o | | | o | o | o |
| o | | | o | o | o |

**25.Negli ultimi 12 mesi, il paziente si è sottoposto a visite specialistiche?**

o Sì

o No ⇒ *Passi alla domanda 27*

**26. A quante visite specialistiche il paziente si è sottoposto negli ultimi 12 mesi?**

*Se qualcuna delle specialità non è elencata, per favore ci specifici quale.*

| | Visite specialistiche | N° di visite in 12 mesi | Costo coperto dal Servizio Sanitario Nazionale (ASL) | | |
|---|---|---|---|---|---|
| | | | Sì | No | Parziale |
| o | Diagnostica genetica | | o | o | o |
| o | Cardiologica | | o | o | o |
| o | Chirurgica | | o | o | o |
| o | Dermatologica | | o | o | o |
| o | Gastrointerologica | | o | o | o |
| o | Endocrinologica | | o | o | o |
| o | Fisioterapica | | o | o | o |
| o | Ginecologica | | o | o | o |
| o | Ematologica | | o | o | o |
| o | Immunologica | | o | o | o |
| o | Logopedistica | | o | o | o |
| o | Nefrologica | | o | o | o |
| o | Pneumologica | | o | o | o |
| o | Neurochirurgica | | o | o | o |
| o | Neurologica | | o | o | o |
| o | Dentistica | | o | o | o |
| o | Oculistica | | o | o | o |
| o | Oncologica | | o | o | o |
| o | Otorinolaringoiatrica | | o | o | o |
| o | Podologica | | o | o | o |
| o | Psicologica | | o | o | o |
| o | Psichiatrica | | o | o | o |
| o | Reumatologica | | o | o | o |
| o | Traumatologica | | o | o | o |
| o | Urologica | | o | o | o |
| o | | | o | o | o |
| o | | | o | o | o |
| o | | | o | o | o |
| o | | | o | o | o |
| o | | | o | o | o |
| o | | | o | o | o |

**27.Negli ultimi 6 mesi, il paziente si è sottoposto a sedute di terapia riabilitativa?**

o Sì

o No ⇒ *Passi alla domanda 29*

**28. A quante sedute di terapia riabilitativa è stato sottoposto il paziente negli ultimi 6 mesi?**

*Se qualcuna delle sedute non è elencata, per favore ci specifici quale.*

| | Sedute di terapia | N° di sedute in 6 mesi | Costo coperto dal Servizio Sanitario Nazionale (ASL) | | |
|---|---|---|---|---|---|
| | | | Sì | No | Parziale |
| o | Fisica | | o | o | o |
| o | Cognitiva | | o | o | o |
| o | Logopedia | | o | o | o |
| o | | | o | o | o |
| o | | | o | o | o |
| o | | | o | o | o |

**29. A quante visite del Medico di Medicina Generale (MMG) / Infermiere / Pronto Soccorso - Servizi di continuità assistenziale il paziente è stato sottoposto negli ultimi 6 mesi?**

o MMG
___ visite presso il suo studio
___ visite a domicilio

o Infermiere
___ visite presso l'ambulatorio
___ visite a domicilio
___ visite presso l'ospedale

o Pronto soccorso - Servizi di continuità assist.
___ accessi al Pronto soccorso ospedaliero
___ visite a domicilio
___ consultazioni telefoniche

**30. Quante volte e per quanti giorni complessivamente il paziente è stato ricoverato in ospedale negli ultimi 12 mesi (specificatamente a causa della sua malattia)?**

n ___ volte

n ___ giornate di degenza in totale

**31. Il paziente ha utilizzato materiale sanitario negli ultimi 6 mesi?**

o Sì

o No ⇒ *Passi alla domanda 34*

**32. Per favore, specifichi il materiale sanitario che il paziente ha dovuto usare negli ultimi 6 mesi.**

*Se qualcuno dei materiali usati non è elencato, per favore ce lo specifichi.*

| | Materiale sanitario | Costo coperto dal Servizio Sanitario Nazionale (ASL) | | |
|---|---|---|---|---|
| | | Sì | No | Parziale |
| o | Panolloni | o | o | o |
| o | Passeggino | o | o | o |
| o | Macchinette per aerosol | o | o | o |
| o | Scarpa ortopedica | o | o | o |
| o | Ausili terapeutici (scivoli, altalene, tappetti, attrezzi per esercici gnnici etc) | o | o | o |
| o | Altro (specificare) | o | o | o |
| o | | o | o | o |
| o | | o | o | o |
| o | | o | o | o |
| o | | o | o | o |
| o | | o | o | o |
| o | | o | o | o |
| o | | o | o | o |
| o | | o | o | o |

**33. Per quali motivi il paziente ha avuto eventuali difficoltà ad ottenere questo materiale sanitario?**

o Mancanza di scorte del prodotto

o Il prodotto è stato ritirato dal mercato

o Il prodotto è stato importato da un altro Paese

o Il prodotto è indicato/prescritto per una patologia diversa da quella del paziente

o Il prodotto è troppo costoso

o Il dosaggio o la confezione disponibile non è appropriata alle necessità del paziente (ad es. non è disponibile il dosaggio necessario per le specifiche caratteristica e condizioni)

o Altre difficoltà (per favore, specificare): ___________________________________

o Non ci sono state difficoltà nell’ottenimento del materiale

**34. Quante volte il paziente ha usato mezzi di trasporto negli ultimi 6 mesi per recarsi, per motivi specificatamente correlati alla sua malattia, presso ambulatori, ospedali, centri di riabilitazione ecc. ecc.?**

| | Tipo di trasporto | N° di volte negli ultimi 6 mesi | Costo coperto dal Servizio Sanitario Nazionale (ASL) | | |
|---|---|---|---|---|---|
| | | | Sì | No | Parziale |
| o | Auto privata | | o | o | o |
| o | Taxi | | o | o | o |
| o | Bus/Treno | | o | o | o |
| o | Aereoplano | | o | o | o |
| o | Mezzi di trasporto di tipo sanitario | | o | o | o |
| o | Ambulanza | | o | o | o |
| o | Automezzi Associazioni di Volontariato (o automezzi di tipo socio/sanitario) | | o | o | o |

**35. Il paziente ha richiesto servizi sanitari e sociali nell'ultimo mese?**

o Sì

o No ⇒ *Passi alla domanda 37*

**36. Indichi i servizi sanitari e sociali che il paziente ha richiesto e avuto, il sistema di finanziamento e gli eventuali motivi per cui non ha avuto i servizi richiesti.**

*Segni i servizi richiesti, il numero di giorni in cui sono stati erogati e la modalità di pagamento.*

**Nel caso abbia richiesto un servizio e non l'abbia ottenuto, segni il numero della motivazione (le possibili motivazioni sono elencate e numerate nella nota con asterisco sotto la tabella).*

**Nell'ultimo mese...**

| Tipi di servizio | A causa degli effetti della malattia è stato necessario il servizio | Le giornate di servizio erogato a seconda delle modalità di finanziamento | | | Ragioni per cui non sono stati erogati i servizi richiesti (*) |
|---|---|---|---|---|---|
| | | Gratuite | Pagamento a proprio carico | Misto (pubblico e privato) | |
| **Nell'ultimo mese...** | | **Nell'ultimo mese...** | | | |
| 1. Teleassistenza | O | ___ giorni | ___ giorni | ___ giorni | |
| 2. Assistenza domiciliare programmata | O | ___ giorni | ___ giorni | ___ giorni | |
| a. sociale (es. accudenti) | O | ___ giorni | ___ giorni | ___ giorni | |
| b. socio-sanitaria | O | ___ giorni | ___ giorni | ___ giorni | |
| c. sanitaria (infermieristica specializzata) | O | ___ giorni | ___ giorni | ___ giorni | |
| 3. Assistenza semiresidenziale | O | ___ giorni | ___ giorni | ___ giorni | |
| a. sociale (centri polifunzionali - Attività culturali, ricreative e per il tempo libero) | O | ___ giorni | ___ giorni | ___ giorni | |
| b. socio-sanitaria (es:centri diurni persone con disabilità, centri occupazionali) | O | ___ giorni | ___ giorni | ___ giorni | |
| c. sanitaria (es. centri di riabilitazione) | O | ___ giorni | ___ giorni | ___ giorni | |
| 4. Assistenza residenziale | O | ___ giorni | ___ giorni | ___ giorni | |
| a. sociale (es. casa famiglia, comunità alloggio) | O | ___ giorni | ___ giorni | ___ giorni | |
| b. socio-sanitaria (es:RSA Residenza sanitaria assistita) | O | ___ giorni | ___ giorni | ___ giorni | |
| c. sanitaria (lungodegenza e servizi riabilitativi, centri riabilitativi) | O | ___ giorni | ___ giorni | ___ giorni | |
| 5. Altro :... | O | ___ giorni | ___ giorni | ___ giorni | |
| 6. Altro :... | O | ___ giorni | ___ giorni | ___ giorni | |
| 7. Altro :... | O | ___ giorni | ___ giorni | ___ giorni | |

**(*) Ragioni: 1 – Lista d'attesa. 2 – Non c'è disponibilità di offerta. 3 – Non c'è disponibilità economica alla compartecipazione al costo. 4 – Non ho i requisiti per accedere. 5 – Altre ragioni.**

**Negli ultimi 6 mesi ...**

| Tipi di servizio | A causa degli effetti della malattia è stato necessario il servizio | Le giornate di servizio erogato a seconda delle modalità di finanziamento | | | Ragioni per cui non sono stati erogati i servizi richiesti (*) |
|---|---|---|---|---|---|
| | | Gratuite | Pagamento a proprio carico | Misto (pubblico e privato) | |
| **Negli ultimi 6 mesi...** | | **Negli ultimi 6 mesi ...** | | | |
| 8. Terapia occupazionale e/o esercizi per svolgere le attività quotidiane (ADLs - Activities of Daily Living) | O | ___ giorni | ___ giorni | ___ giorni | |
| 9. Informazioni / Consulenze / Valutazioni | O | ___ giorni | ___ giorni | ___ giorni | |
| 10. Assistenza psicologica ai familiari | O | ___ giorni | ___ giorni | ___ giorni | |
| 11. Servizi di sollievo: soggiorni temporanei in strutture residenziali | O | ___ giorni | ___ giorni | ___ giorni | |
| 12. Servizi da parte di interpreti del linguaggio dei segni | O | ___ giorni | ___ giorni | ___ giorni | |
| 13. Altri sistemi di comunicazione alternativi | O | ___ giorni | ___ giorni | ___ giorni | |
| 14. Centri residenziali | O | ___ giorni | ___ giorni | ___ giorni | |
| 15. Turismo e idroterapia per persone disabili | O | ___ giorni | ___ giorni | ___ giorni | |
| 16. Orientamento / preparazione al lavoro | O | ___ giorni | ___ giorni | ___ giorni | |
| 17. Musicoterapia | O | ___ giorni | ___ giorni | ___ giorni | |
| 18. Ippoterapia | O | ___ giorni | ___ giorni | ___ giorni | |
| 19. Nuoto | O | ___ giorni | ___ giorni | ___ giorni | |
| 20. Altro:... | O | ___ giorni | ___ giorni | ___ giorni | |
| 21. Altro:... | O | ___ giorni | ___ giorni | ___ giorni | |

**(*) Ragioni: 1 – Lista d'attesa. 2 – Non c'è disponibilità di offerta. 3 – Non c'è disponibilità economica alla compartecipazione al costo. 4 – Non ho i requisiti per accedere. 5 – Altre ragioni.**

**37.E' soddisfatto dell'assistenza sanitaria ricevuta dal paziente specificatamente alla malattia?**

Per favore indichi il grado della sua soddisfazione in una scala di 1 a 10.

**1 2 3 4 5 6 7 8 9 10**

Per nulla soddisfatto .......................................................... Molto soddisfatto

Si prega di riempire anche i seguenti questionari:

- **QUESTIONARIO SULLA SALUTE DEL PAZIENTE (EQ-5D-5L)**
- **QUESTIONARIO SULL'AUTONOMIA DEL PAZIENTE (WHODAS 2.0)**
- **QUESTIONARIO PER LA PERSONA CHE ASSISTE IL MALATO**
- **QUESTIONARIO SULLA SALUTE DELLA PERSONA CHE ASSISTE IL MALATO (EQ-5D-5L)**
