# Supplementary material for: Social Economic Costs, Health-Related Quality of Life and Disability in Patients with Cri Du Chat Syndrome
Source: Int J Environ Res Public Health. 2020 Aug 17;17(16):5951. doi: 10.3390/ijerph17165951 (PMC7459640; doi:10.3390/ijerph17165951)
Supplement: Supplementary file 1 [file ijerph-17-05951-s001.zip › S2.pdf]

QUESTIONARIO SULL'AUTONOMIA DEL PAZIENTE (WHODAS 2.0)

Questo questionario indaga sulle <u>difficoltà dovute a condizioni di salute</u> sperimentate del paziente riguardo al quale lei risponde nel suo ruolo di familiare, congiunto, amico o assistente. Le condizioni di salute includono infermità o malattie, altri problemi di salute che possono essere di breve o lunga durata, lesioni, problemi mentali o emotivi e problemi di alcool o droghe.

Ripensi agli <u>ultimi 30 giorni</u>, al meglio delle sue conoscenze, e risponda a queste domande pensando a quanta difficoltà ha avuto il paziente mentre effettuava le seguenti attività.
Per ciascuna domanda <u>indichi solo una risposta</u>

* 42. **COMPRENDERE E COMUNICARE**
Negli ultimi 30 giorni, quanta difficoltà il paziente ha avuto nel

	Nessuna	Lieve	Media	Grave	Estrema o non può farlo
(D1.1) Concentrarsi nel fare qualcosa per 10 minuti?	○	○	○	○	○
(D1.2) Ricordare di fare cose importanti?	○	○	○	○	○
(D1.3) Analizzare e trovare soluzioni ai problemi quotidiani?	○	○	○	○	○
(D1.4) Apprendere un nuovo compito, per esempio imparare come raggiungere un posto nuovo?	○	○	○	○	○
(D1.5) In genere capire cosa dice la gente?	○	○	○	○	○
(D1.6) Iniziare e portare avanti una conversazione?	○	○	○	○	○

* 43. **SPOSTAMENTI**
negli ultimi 30 giorni, quanta difficoltà il paziente ha avuto nel:

	Nessuna	Lieve	Media	Grave	Estrema o non può farlo
(D2.1) Rimanere in piedi per lunghi periodi, tipo 30 minuti?	○	○	○	○	○
(D2.2) Alzarsi dalla posizione seduta?	○	○	○	○	○
(D2.3) Muoversi dentro casa?	○	○	○	○	○
(D2.4) Uscire di casa?	○	○	○	○	○
(d2.5) Camminare per una lunga distanza tipo un chilometro (o equivalente)?	○	○	○	○	○

* 44. **CURA PERSONALE**
negli ultimi 30 giorni, quanta difficoltà il paziente ha avuto nel:

	Nessuna	Lieve	Media	Grave	Estrema o non può farlo
(D3.1) Lavarsi l'intero corpo?	○	○	○	○	○
(D3.2) Vestirsi?	○	○	○	○	○
(D3.3) Nutrirsi?	○	○	○	○	○
(D3.4) Rimanere da solo per pochi giorni?	○	○	○	○	○

* 45. **INTERAGIRE CON LE PERSONE**
negli ultimi 30 giorni, quanta difficoltà il paziente ha avuto nel:

	Nessuna	Lieve	Media	Grave	Estrema o non può farlo
(D4.1) Interagire con persone che non conosce?	○	○	○	○	○
(D4.2) Mantenere un'amicizia?	○	○	○	○	○
(D4.3) Interagire con persone vicine?	○	○	○	○	○
(D4.4) Fare nuove amicizie?	○	○	○	○	○
(D4.5) Attività sessuale?	○	○	○	○	○

* 46. **ATTIVITA' QUOTIDIANE**
negli ultimi 30 giorni, quanta difficoltà il paziente ha avuto nel:

	Nessuna	Lieve	Media	Grave	Estrema o non può farlo
(D5.1) Adempiere alle sue responsabilità domestiche?	◯	◯	◯	◯	◯
(D5.2) Fare bene le più importanti cose di casa?	◯	◯	◯	◯	◯
(D5.3) Finire tutto il lavoro domestico che doveva fare?	◯	◯	◯	◯	◯
(D5.4) Fare tutto il lavoro domestico tanto velocemente quanto necessitava?	◯	◯	◯	◯	◯

* 47. **Lavora (pagato, non-pagato, lavoratore autonomo) o va a scuola?**

- Si
- No

* 48. **Negli ultimi 30 giorni, quanta difficoltà il paziente ha avuto nel:**

	Nessuna	Lieve	Media	Grave	Estrema o non può farlo
(D5.5) Suo lavoro/studio quotidiano?	◯	◯	◯	◯	◯
(D5.6) Fare bene i più importanti compiti lavorativi/di studio?	◯	◯	◯	◯	◯
(D5.7) Finire tutto il lavoro da fare?	◯	◯	◯	◯	◯
(D5.8) Finire il lavoro tanto velocemente quanto necessitava?	◯	◯	◯	◯	◯

* 49. **PARTECIPAZIONE NELLA SOCIETA'**
Negli ultimi 30 giorni:

	Nessuna	Lieve	Media	Grave	Estrema o non può farlo
(D6.1) Quanti problemi ha incontrato nel partecipare come chiunque altro, ad attività comunitarie (per esempio, feste, attività religiose o altro)	○	○	○	○	○
(D6.2) Quanti problemi ha incontrato a causa di barriere architettoniche o ostacoli nel mondo che la circonda?	○	○	○	○	○
(D.6.3) Quanti problemi ha incontrato nel vivere con dignità a causa degli atteggiamenti e delle azioni altrui?	○	○	○	○	○
(D6.4) Quanto tempo ha dedicato alla sua condizione di salute o alle sue conseguenze?	○	○	○	○	○
(D6.5) Quanto è stato coinvolto emotivamente dalle sue condizioni di salute?	○	○	○	○	○
(D6.6) Quanto le sue condizioni di salute hanno inciso sulle risorse finanziarie e quelle della sua famiglia?	○	○	○	○	○
(D6.7) Quanti problemi ha avuto la sua famiglia a causa dei suoi problemi di salute?	○	○	○	○	○
(D6.8) Quanti problemi ha avuto nel fare cose da solo per relax o piacere?	○	○	○	○	○

* 50. **Indichi il numero dei giorni per le seguenti attività:**

(H1) Complessivamente, negli ultimi 30 giorni, per quanti giorni il paziente ha avuto queste difficoltà?

(H2) Negli ultimi 30 giorni, per quanti giorni è stato/a completamente inabile a condurre le sue abituali attività o lavoro a causa di una qualunque condizione di salute?

(H3) Negli ultimi 30 giorni, non contando i giorni in cui lei è stato/a completamente inabile, per quanti giorni il paziente ha diminuito o ridotto le sue abituali attività o lavoro a causa di una qualsiasi condizione di salute?
