# Supplementary material for: Social Economic Costs, Health-Related Quality of Life and Disability in Patients with Cri Du Chat Syndrome
Source: Int J Environ Res Public Health. 2020 Aug 17;17(16):5951. doi: 10.3390/ijerph17165951 (PMC7459640; doi:10.3390/ijerph17165951)
Supplement: Supplementary file 1 [file ijerph-17-05951-s001.zip › S3.pdf]

QUESTIONARIO PER LA PERSONA CHE ASSISTE IL MALATO (Caregiver)

1. **Età della persona che assiste** ____ anni
2. **Sesso** o Femmina o Maschio
3. **Provincia di residenza** _______________
4. **Stato civile**
   - o Celibe
   - o Coniugato/a o convivente
   - o Divorziato/a
   - o Separata/o
   - o Vedovo/a
5. **Da quando ha iniziato a prendersi cura del paziente?** ______anni ______mesi
6. **Qual è la sua attuale situazione lavorativa?**
   - o Occupato
   - o Ritirato dalla sua attività professionale ⇒ Passi alla domanda 8
   - o pensionato ⇒ Passi alla domanda 9
   - o Casalinga ⇒ Passi alla domanda 9
   - o Altro (studente, disoccupato, ecc. ecc,.) ⇒ Passi alla domanda 9
7. **Problemi relativi allo stato occupazionale (si risponde solo se la persona che assiste è ancora nel mercato del lavoro, ossia occupato o non occupato da meno di 12 mesi):**
   a. **Negli ultimi 12 mesi, il prendersi cura del paziente, in seguito alla sua patologia, ha comportato dei problemi rispetto al suo lavoro?**
      o Sì o No ⇒ Si passi alla domanda 9
   b. **Se sì, si prega di specificare se negli ultimi 12 mesi:**
      - o Ho dovuto chiedere ____ giorni di permesso
      - o Ho lavorato ____ ore in meno al giorno per _____ giorni
      - o Ho lavorato sempre ____ ore in meno al giorno
      - o Non lavoro meno ore al giorno, ma ho difficoltà a gestire l'orario di lavoro richiesto.
      - o Altri problemi (si prega di specificare): ________________________________

⇒ Si passi alla domanda 9

8. **La persona che assiste ha risposto che si è ritirato dalla sua attività professionale**
   a. **Ha deciso di ritirarsi prima del previsto per assistere il paziente?**
      o Sì o No ⇒ Si passi alla domanda 9
   b. **Se sì, si prega di specificare:**
      o Mi sono ritirato all'età di ____ anni.

Profilo del ruolo svolto come PERSONA PRINCIPALE CHE ASSISTE IL MALATO…

9a. Quanto tempo dedica, in una GIORNATA tipo, a ciascuna delle attività richieste dalla malattia del paziente e di seguito elencate?
Per favore specifichi approssimativamente il tempo impiegato quotidianamente per svolgere ciascuna delle seguenti attività:

Igiene di base, vestizione o cambio	....	ore	....	minuti	al giorno
Fare la doccia o il bagno	....	ore	....	minuti	al giorno
Pasti (nutrimento)	....	ore	....	minuti	al giorno
Aiuto a muoversi	....	ore	....	minuti	al giorno
Cucinare e preparare i pasti	....	ore	....	minuti	al giorno
Somministrare i farmaci	....	ore	....	minuti	al giorno

9b. Quanto tempo dedica, in una SETTIMANA tipo, a ciascuna delle attività richieste dalla malattia del paziente e di seguito elencate?
Per favore specifichi approssimativamente il tempo impiegato settimanalmente per svolgere ciascuna delle seguenti attività:

Occupazioni domestiche (pulizia, lavaggio del guardaroba, ecc. ecc....)	....	ore	....	minuti	al giorno
Viaggi	....	ore	....	minuti	al giorno
Nel fare la spesa	....	ore	....	minuti	al giorno
Nel gestire aspetti finanziari, amministrativi o legali	....	ore	....	minuti	al giorno
In attività sociali e di svago	....	ore	....	minuti	al giorno
Nel controllare e coordinare (cadute)	....	ore	....	minuti	al giorno
Nelle terapie riabilitative	....	ore	....	minuti	al giorno
Altro	....	ore	....	minuti	al giorno

Profilo del ruolo svolto dalle ALTRE PERSONE CHE ASSISTONO (ad esempio, il resto della famiglia, volontari ed amici)

10a. Quanto tempo dedicano, in una GIORNATA tipo, le altre persone che assistono a ciascuna delle attività richieste dalla malattia del paziente e di seguito elencate?
Per favore specifichi approssimativamente il tempo impiegato settimanalmente per svolgere ciascuna delle seguenti attività:

Igiene di base, vestizione o cambio	....	ore	....	minuti	al giorno
Fare la doccia o il bagno	....	ore	....	minuti	al giorno
Pasti (nutrimento)	....	ore	....	minuti	al giorno
Aiuto a muoversi	....	ore	....	minuti	al giorno
Cucinare e preparare i pasti	....	ore	....	minuti	al giorno
Somministrare i farmaci	....	ore	....	minuti	al giorno

10b. Quanto tempo dedicano, in una SETTIMANA tipo, le altre persone che assistono a ciascuna delle attività richieste dalla malattia del paziente e di seguito elencate?
Per favore specifichi approssimativamente il tempo impiegato settimanalmente per svolgere ciascuna delle seguenti attività:

Occupazioni domestiche (pulizia, lavaggio del guardaroba, ecc. ecc....)	....	ore	....	minuti	al giorno
Viaggi	....	ore	....	minuti	al giorno
Nel fare la spesa	....	ore	....	minuti	al giorno
Nel gestire aspetti finanziari, amministrativi o legali	....	ore	....	minuti	al giorno

In attività sociali e di svago	....	ore	....	minuti	al giorno
Cotrollare e coordinare (cadute)	....	ore	....	minuti	al giorno
Nelle terapie riabilitative	....	ore	....	minuti	al giorno
Altro	....	ore	....	minuti	al giorno
