# Supplementary material for: Social Economic Costs, Health-Related Quality of Life and Disability in Patients with Cri Du Chat Syndrome
Source: Int J Environ Res Public Health. 2020 Aug 17;17(16):5951. doi: 10.3390/ijerph17165951 (PMC7459640; doi:10.3390/ijerph17165951)
Supplement: Supplementary file 1 [file ijerph-17-05951-s001.zip › S4.pdf]

Questionario sulla Salute del Paziente

Italy(Italian)© 2.6196898440588E-30515 EuroQol Research Foundation. EQ-5D™ is a trade mark of the EuroQol Research Foundation

Sotto ciascun argomento, faccia una crocetta sulla casella (UNA SOLA) che a suo avviso descrive meglio la salute del paziente OGGI.

CAPACITÀ DI MOVIMENTO

Nessuna difficoltà nel camminare ❑
Lievi difficoltà nel camminare ❑
Moderate difficoltà nel camminare ❑
Gravi difficoltà nel camminare ❑
Non in grado di camminare ❑

CURA DELLA PERSONA

Nessuna difficoltà nel lavarsi o vestirsi ❑
Lievi difficoltà nel lavarsi o vestirsi ❑
Moderate difficoltà nel lavarsi o vestirsi ❑
Gravi difficoltà nel lavarsi o vestirsi ❑
Non in grado di lavarsi o vestirsi ❑

ATTIVITÀ ABITUALI (*per es. lavoro, studio, lavori domestici, attività familiari o di svago)*

Nessuna difficoltà nello svolgimento delle attività abituali ❑
Lievi difficoltà nello svolgimento delle attività abituali ❑
Moderate difficoltà nello svolgimento delle attività abituali ❑
Gravi difficoltà nello svolgimento delle attività abituali ❑
Non in grado di svolgere le attività abituali ❑

DOLORE O FASTIDIO

Nessun dolore o fastidio ❑
Lieve dolore o fastidio ❑
Moderato dolore o fastidio ❑
Grave dolore o fastidio ❑
Estremo dolore o fastidio ❑

ANSIA O DEPRESSIONE

Per niente ansioso/a o depresso/a ❑
Lievemente ansioso/a o depresso/a ❑
Moderatamente ansioso/a o depresso/a ❑
Gravemente ansioso/a o depresso/a ❑
Estremamente ansioso/a o depresso/a ❑

Italy(Italian)© 2.6196898440588E-30515 EuroQol Research Foundation. EQ-5D™ is a trade mark of the EuroQol Research Foundation

- **Vorremmo sapere quanto ritiene sia buona o cattiva la salute del paziente OGGI.**
- **Questa è una scala numerata che va da 0 a 100.**
- **100 rappresenta la <u>migliore</u> salute che può immaginare.**
  0 rappresenta la <u>peggiore</u> salute che può immaginare.
- **Segni una X sul punto della scala per indicare quanto ritiene sia buona o cattiva la salute del soggetto OGGI.**
- **Poi, scriva nella casella qui sotto il numero che ha segnato sulla scala numerata.**

LA SALUTE DEL SOGGETTO OGGI [ ]

La migliore salute che può immaginare

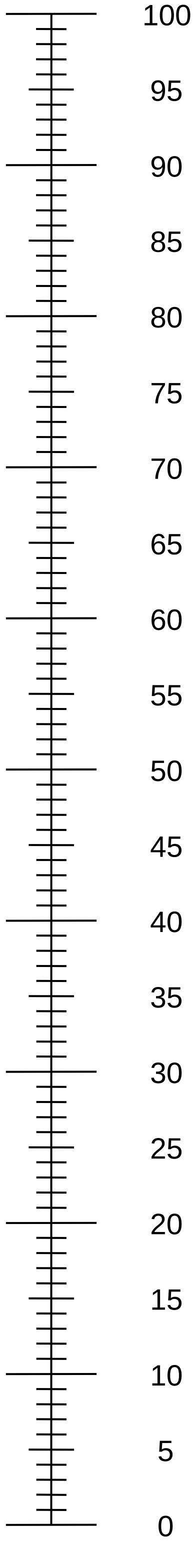

La peggiore salute che può immaginare

Italy(Italian)© 2.6196898440588E-30515 EuroQol Research Foundation. EQ-5D™ is a trade mark of the EuroQol Research Foundation
